# *Ipomea batatas* Leaf Powder from Cameroon: Antioxidant Activity and Antihyperlipidemic Effect in Rats Fed with a High-Fat Diet

**DOI:** 10.1155/2021/5539878

**Published:** 2021-06-10

**Authors:** Marcéline Joëlle Mbouche Fanmoe, Léopold Tatsadjieu Ngoune, Robert Ndjouenkeu

**Affiliations:** ^1^Department of Food Science and Nutrition, National School of Agro-Industrial Sciences, University of Ngaoundere, P.O. Box 455, Ngaoundere, Cameroon; ^2^Department of Biochemistry, Faculty of science, University of Yaounde I, P.O. Box 812, Yaounde, Cameroon; ^3^Department of Food Engineering and Quality Control, University Institute of Technology, University of Ngaoundere, P.O. Box 454, Ngaoundere, Cameroon

## Abstract

The present study consists of analyzing the phytochemical composition of *Ipomoea batatas* leaf powders and evaluating their antihyperlipidemic effect on rats receiving a high-fat diet. *Ipomoea batatas* leaves were collected from four agroecological areas of Cameroon, and powders were obtained after washing, drying, grinding, and sieving. Standard analytical methods were used to determine the phytochemical composition of two varieties (IRAD-tib1yellow-V1 and IRAD-1112white-V2) from North Z1, Adamawa Z2, West Z3, Center Z4. The effect of *I. batatas* leaf powder on lipid metabolism was assessed *in vivo* by feeding different groups of rats with a high-fat diet supplemented with 5 and 10% of *I. batatas* leaf powder during 30 days. At the end of the experimentation, total cholesterols, triglycerides, LDL- (low-density lipoprotein-) cholesterol, HDL- (High-density lipoprotein-) cholesterol, ASAT (aspartate aminotransferase), ALAT (alanine aminotransferase), and creatinine were measured using commercial enzymatic kits (Spinreact, Spain). The results of phytochemical analysis of *I. batatas* leaf powders revealed that the total phenol content ranged from 660.173 mg GAE/100 gDW (Z1V2) to 657.76 mg GAE/100 gDW (Z3V2), the flavonoids content ranged from 282.25 mgEC/100 gDW (Z3V1) to 325.05 mgEC/100 gDW (Z4V2), and the anthraquinone content ranged from 324.05 mg/100 gDW (Z3V2) to 326.72 mg/100 gDW (Z4V2). The total antioxidant capacity ranged from 19.00 (Z1V1) to 23.48 mg AAE/gDW (Z3V2), while the IC_50_ ranged from 1.58 mg/mL (Z1V1) to 3.08 mg/mL (Z3V2). Rats fed a high-fat diet and supplemented with 5 and 10% of *I. batatas* leaf powder showed a significant (*p* < 0.05) reduction in body weight compared to the control with a reduction rate ranging from 6 to 10%. The consumption of *I. batatas* leaf powder for 30 days significantly (*p* < 0.05) reduced serum total cholesterol, LDL-cholesterol, triglycerides, ALAT, and creatinine level. These results suggest the use of *I. batatas* leaves as a phytomedicine in the treatment of cardiovascular diseases.

## 1. Introduction

Nowadays, dietary habits have changed and the tendency is much more oriented towards traditional foods based on vegetables very rich in antioxidants to prevent cardiovascular diseases [1]. Among vegetables, the leaves of *Ipomea batatas* (sweet potato) are an essential part of the human diet, providing the body with vitamins, minerals, proteins, some hormone precursors, and a high content of phytochemicals endowed with beneficial effects on health [2]. In Japan, the leaves of *I. batatas* have been reported to be an excellent source of anthocyanins and many other bioactive compounds [3]. In Brazil and Malaysia, some studies on the leaves of *I. batatas* have focused on its antioxidant capacity due to its high content of flavonoids, *β* carotene, anthocyanins, and phenols [4, 5]. Therefore, the presence of all these compounds mentioned could be the reason *I. batatas* leaves are used in traditional medicine (anaemia, antiviral, and worm). In addition to their antidiabetic and antiviral properties, *Ipomoea batatas* leaves have been used to treat infectious diseases in Brazil [6]. Phytochemical compounds protect the body against degenerative diseases such as cancer and atherosclerosis caused by free radicals. Because of their potential as a bioactive compound, the use of *I. batatas* leaves has also been considered for the management of hyperlipidemia. Hyperlipidemia is a physiological disturbance characterized by an abnormal increase in cholesterol levels or blood lipoproteins [7]. It is the main cause of cardiovascular diseases, such as ischemic cerebrovascular disease, coronary heart disease, and peripheral vascular disease [8, 9]. On the other hand, hyperlipidemia is the main cause of mortality (about 12 million people) and morbidity in the world [10]. Many lipid-lowering drugs (fibers, sequestering bile acids, etc.) are used to regulate lipid metabolism by various mechanisms, but due to their synthetic nature, they are also known for their negative effects on health, including hyperuricemia, diarrhea, nausea, severe muscle damage (myopathy), gastric irritation, hot flashes, dry skin, and deregulation of liver function [11]. A number of plants have been traditionally used against various cardiovascular diseases and to control hyperlipidemia, among which the leaves of *I. batatas* [6, 12]. Some surveys conducted in some localities in Cameroon have reported their use in traditional medicine as a slimming plant. This research therefore aims to quantify the phytochemical compounds of *Ipomoea batatas* leaves collected in different agroecological zones of Cameroon and to study their antihyperlipidemic activity in rats fed a high-fat diet.

## 2. Materials and Methods

### 2.1. Plant Material and Preparation of Ipomea Batatas Leaf Powder

Fresh leaves of two (2) varieties, yellow *I. batatas* IRAD-Tib1(V1) and white *I. batatas* IRAD-1112 (V2), were collected to approximately 20 cm starting from the apical pole in four areas of study: Sudano-Sahellian (Z1, North: Garoua), Guinea savannah high zone (Z2, Adamawa: Ngaoundere), Western highland zone (Z3, West: Foumban), and the rain forest with two modes (Z4, Center: Bafia) and were identified by Dr. Nérée Onguéné, plant taxonomist at the Agricultural Research Institute for Development of Yaounde (IRAD-Yaounde, Cameroon), and Mr. MBONOMO Jean Moise, Technician of Institute of Agronomic Research for Development (IRAD-Wakwa, Ngaoundere-Cameroon).

Leaves were washed and dried in the oven at 45°C for 48 hours and crushed with a moulin through a sieve mesh of 500 *μ*m to produce fine leaf powder. The powders were stored in plastic packaging and hermetically sealed for further analysis.

### 2.2. Quantitative Estimation of Fiber and Phytochemical Compounds

#### 2.2.1. Fiber Content

The Wolff [13] method was used to determine the crude fiber content in leaf powders. 2 g of sample was boiled for 30 min under a reflux condenser in 0.25 N sulphuric acid solution. The mixture was filtered, and then, the residue was rinsed and boiled again for 30 min in a 0.3 N NaOH solution. Then, the filtered and rinsed sample was dried for 8 hours at 105°C. After cooling, the residue was weighed and then incinerated in a muffle furnace at 550°C for 3 hours. The crude fiber content was expressed as a percentage of the dry weight (DW) of the sample.

#### 2.2.2. Total Phenol and Flavonoid Content

The method described by Hsu [14] was used for the extraction of phenolic compounds. Eighty mL of methanol (80%) was introduced into a beaker containing 5 g of leaf powder, and the mixture was homogenized and left to macerate for 12 hours. The mixture was then centrifuged (5000 rpm, 10 min, 4°C) and filtered through Whatman N°1 filter paper. The filtrate was then made up with methanol (80%) to 100 mL to serve as a stock solution (50 mg/mL). Then, the total phenol and flavonoid content were determined using the protocol described by Sokamte et al. [15].

#### 2.2.3. Proanthocyanidins Content

The vanillin method described by Broadhurst and Jones [16] was used for the determination of proanthocyanidin content. 25 *μ*l of the sample (stock solution) was mixed with 125 *μ*l of vanillin reagent (4% in methanol) and 60 *μ*l of concentrated hydrochloric acid (35%). Absorbance was measured at 500 nm after 15 min incubation in the dark and at room temperature. The calibration curve was drawn with catechin (0-1 mg/mL). The proanthocyanidin content was expressed in mg catechin equivalent/g dry weight (mg CE/g DW).

#### 2.2.4. Anthraquinone Content

For the determination of the anthraquinone content, the stock solution was evaporated under vacuum using a rotary evaporator to obtain the dry extract. Then, 1 g of extract was mixed with 30 mL of distilled water and boiled for 15 min under a reflux condenser. The analysis procedure was following with the steps described in the protocol of Sakulpanich and Gritsanapan [17], and the results were expressed as mg of hydroxyanthracene derivatives/100 g DW.

### 2.3. Determination of Antioxidant Activities

The antioxidant activity of the samples was evaluated using three tests: the DPPH test (1, 1-Diphenyl-2-Picrylhydrazyl test), the FRAP (Ferric Reducing Antioxidant Power) test, and the TAC (total antioxidant capacity) test. The analysis procedures were following the steps described in our previous publication [18]. For the DPPH test, the results were expressed in IC_50_ (concentration of the extract that allows 50% of the DPPH free radical to be trapped) and were calculated using the exponential regression equation [15].

### 2.4. Experimental Animals

36 male Wistar albino rats of around 3 months, weighing around 250 g from the Laboratory of Nutrition, Faculty of Sciences, University of Yaounde I, Cameroon, were used for the *in vitro* study. Before the study, the rats were weighed, divided into 6 groups of 6 rats, housed in a single cage, and acclimatized for one week with free access to tap water and food. The animals were kept in an environment of 25 ± 2°C with a relative humidity of 55 ± 5% under a cycle of 12 h light and 12 h dark. Procedures used in the study were approved by the Animal Ethics Committee of the Laboratory of Nutrition of the University of Yaounde I. The trial was registered with reference number: No. FW-IRB00001954.

### 2.5. Diet Administration and Blood Collection

The standard and high-fat diets used to feed animals were formulated as proposed by Murase et al. [19] with slight modification (Table 1). The rats were divided into 6 groups of 6 rats each. The negative control standard diet (TN-STD) group received *ad libitum* standard or normal diet. The positive control high-fat diet (TP-HFD) group received *ad libitum* high-fat diet. The TP-HFD-Z2V2 5% group, the TP-HFD-Z2V2 10% group, the TP-HFD-Z3V2 5% group, and the TP-HFD-Z3V2 10% group, fed *ad libitum* with a high-fat diet supplemented with doses of 5 and 10% (*w*/*w*) of *I. batatas* leaf powder from Zone 2/Varity 2 and Zone 3/Variety 2, respectively. The dose of dietary herbal powder supplementation was fixed on the basis of previous studies found in the literature [20, 21]. The experiment was carried out for 30 days, after which the animals were fasted for 12 hours and anaesthetized with ether. The blood was withdrawn from the jugular vein and introduced into dry tubes (without anticoagulant) and then centrifuged (1500 rpm; 10 min) for collection of serum from which subsequent analyses were carried out to assess lipid profile, liver function, and cardiac injury.

### 2.6. Biochemical Assay

#### 2.6.1. Lipid Profile

The levels of total cholesterol, HDL-cholesterol, and LDL-cholesterol in serum were measured using commercial enzymatic kits [22]. Triglycerides were assayed by the GPO-PAP (Glycerol 3 phosphate oxidase—4 Amino-antipyrine) colorimetric method using Human SU-TRIMR 10720P kit (Human, Germany). The triglycerides in the sample were hydrolysed using a set of microbial lipases to form glycerol and fatty acids [23]. The atherogenic index was calculated following the formula described by Bo et al. [24].

#### 2.6.2. Evaluation of Hepatic and Cardiac Function

Hepatic function was assessed by the determination of Alanine Aminotransferase (ALAT) and Aspartate Aminotransferase (ASAT) activities using the spectrophotometer based on the protocols of commercial kits [25]. The results were expressed as IU/L.

The creatinine content was determined using a spectrophotometer working at 520 nm with the Human Su-Crea 10052 kit [26], to know the degree of cardiac injury.

### 2.7. Statistical Analysis

Analyses were carried out in triplicate and data were subjected to analysis of variance to test the significance between treatments. Additionally, differences between means were tested using the Duncan Multiple Range test using Statgraphics Centurion XVI Software version 16.1.18 (StatPoint Technologies, Inc.), and the values were considered statistically different at a risk *p* < 0.05. Regression analysis was performed using SigmaPlot 12 version 12.5.0.38 (Systat Software, Inc.).

## 3. Results and Discussion

### 3.1. Fiber Content

Fiber is the nondigestible polysaccharide residues of plants, and means of total fiber ranged from 8.15 ± 0.02 g/100 gDW (sample Z4V1) to 12.42 ± 0.09 g/100 gDW (sample Z1V2) (Table 2). A significant (*p* < 0.05) difference was observed between the samples, regardless of the origin of the harvest or the variety studied. The results obtained in this study are similar to those of Sun et al. [27]. They found a fiber content of 11.33 g/100 gDW in sweet potato leaf powder from China. As reported by Kurata et al. [20], sweet potato leaves are high in dietary fiber.

### 3.2. Total Phenolics, Flavonoids, Proanthocyanidins, and Anthraquinones Content

Table 2 shows the bioactive compound contents of two varieties of *Ipomea batatas* leaf powder collected from the four localities. As can be seen in this table, the levels of total phenol, proanthocyanidins, and anthraquinones are very similar, regardless of the variety or origin of the plant. However, significant differences (*p* < 0.05) were observed for total phenol and proanthocyanidin contents. The total phenol content varies from 657.76 ± 0.31 gGAE/100 gDW to 660.10 ± 1.00 gGAE/100 gDW, and the proanthocyanidins content varies from 175.27 ± 0.05 gGAE/100 gDW to 176.04 ± 0.06 mgCE/100 g DW. The highest total phenol content was recorded in the Z4V2 sample, while the lowest value was recorded in the Z3V1 sample. On the other hand, the highest proanthocyanidin content was recorded in sample Z1V2, while the lowest value was recorded in sample Z2V1. No significant differences (*p* > 0.05) were observed in the anthraquinone content of the different sweet potato leaf samples. The average levels ranged from 324.05 ± 2.00 to 326.72 ± 2.52 mg/100 gDW. For the total flavonoid content, the variety of the plant has a significant influence on the values recorded in the sweet potato leaves since the lowest contents (282.25 ± 0.12 to 289.13 ± 0.96 mgCE/100 gDW) were observed in variety 1, while the highest contents (323.22 ± 0.20 to 325.05 ± 0.00 mgCE/100 gDW) were observed in varieties 2. Phytochemical compounds such as total phenols and flavonoids have also been detected in the leaves of harvested sweet potatoes in Ethiopia [28] and China [29]. The total phenol content values observed in this study are very high compared to those found by Sun et al. [27] in sweet potato leaves harvested in China (120 mg/100 gDW). In contrast, high levels of total phenols (3680-4670 mg/100 gDW) were found in the leaves of three sweet potato varieties harvested in the USA [30]. These authors suggested that anthocyanins are predominantly present in the extracts studied. Factors that may be responsible for the observed differences in the phytochemical composition of sweet potato leaves are variety, harvest season, growing conditions, and age of the plant as reported by Sokamte et al. [15]. Total phenolic compounds have been lauded for their beneficial effects in the fight against chronic diseases like atherosclerosis, cardiovascular, and diabetes [31]. Moreover, as suggested by Groppo et al. [6], sweet potato leaf was endowed with a potential medicinal use because of its high content of phenolic substances (e.g., tocopherols, tannins, phenolic acids, and flavonoids).

### 3.3. Total Antioxidant Capacity (TAC), Ferric Reducing Antioxidant Power (FRAP), and 1,1-Diphenyl-2-Picrylhydrazyl (DPPH) Assay

The antioxidant activity of the different samples was compared after estimation of the total antioxidant capacity (TAC) and Ferric reducing antioxidant power (FRAP) values in AAE/g DW, which are presented in Table 2. It can be seen from this table that these TAC values differ significantly from one sample to another, with the Z3V2 sample which had the highest activity (23.48 ± 2.75 mg AAE/g DW), followed by the Z2V2 sample (23.02 ± 1.17 mg AAE/g DW). For the FRAP test, all samples of variety 1 showed the lowest activity with mean values ranging from 3.02 ± 0.13 mg AAE/g DW to 3.29 ± 0.27 mg AAE/g DW. No significant difference was observed for these FRAP values, while those of the samples of variety 2 were significantly different. Sample Z3V2 recorded the highest activity (4.10 ± 0.09 mg AAE/g DW). The ability of these samples to trap free radicals was also evaluated using the 1,1-diphenyl-2-picrylhydrazyl (DPPH) test, and the results are expressed in IC_50_. Like the FRAP test, the antiradical capacity of these samples follows the same pattern, as no significant differences were observed for variety 1 (2.90 ± 0.04 to 3.08 ± 0.08 mg/mL, for Z1V1 and Z4V1 samples, respectively). In addition, the highest antiradical activity was recorded for the Z3V2 sample with the lowest IC_50_ value (1.58 ± 0.02 mg/mL). The phenolic compound is considered as the major contributor to the total antioxidant capacity [32]. The high antiradical activity of *I. batatas* leaf powder could explain their use in the traditional treatment of some diseases. Further, considering their antioxidant capacity, Z2V2 and Z3V2 samples could be used to manage lipid profile such as lowering the effect on LDL-cholesterol and increase HDL-cholesterol levels.

### 3.4. Principal Component Analysis

**Figure 1:**
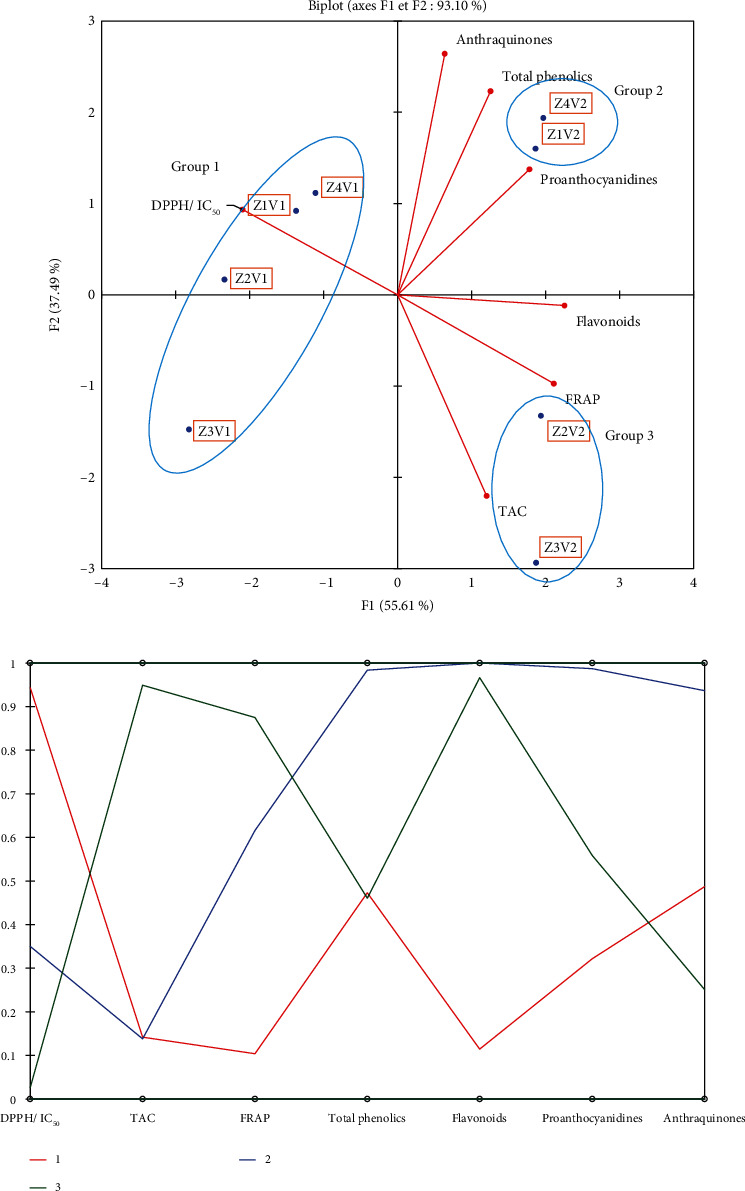
Distribution of *Ipomea batatas* leaf powder and measured parameters on the principal component analysis F1∗F2 axis plane (a). Profiles of the different groups of *I. batatas* leaves (b). (Legend. Z1V1: yellow *I. batatas* IRAD-Tib1 collected from Garoua; Z2V1: yellow *I. batatas* IRAD-Tib1 collected from Ngaoundere; Z3V1: yellow *I. batatas* IRAD-Tib1 collected from Foumban; Z4V1: yellow *I. batatas* IRAD-Tib1 collected from Bafia; Z1V2: white *I. batatas* IRAD-1112 collected from Garoua; Z2V2: white *I. batatas* IRAD-1112 collected from Ngaoundere; Z3V2: white *I. batatas* IRAD-1112 collected from Foumban; Z4V2: white *I. batatas* IRAD-1112 collected from Bafia; 1: group 1; 2: group 2; 3: group 3).

Principal component analysis (PCA) was carried out to group *Ipomea batatas* leaf powders according to similarities or differences in their bioactive compounds and their antioxidant activities. Additionally, the correlations between all measured parameters were revealed after performing the principal component analysis. From Figure 1(a), it can be observed that 93.10% of the total variability in the data are explained by the principal components F1xF2. The first principal component (F1) represents 55.61% of the variation between variables, while the second principal component (F2) represents 37.49%. Tree groups can be observed from this figure, and the characteristics of the different groups obtained are shown in Figure 1(b). In this figure, it can be seen that group 1, represented by samples Z3V1, Z2V1, Z1V1, and Z4V1, is characterized by a lower content of biological compounds and a lower antioxidant activity. Group 2, represented by samples Z1V2 and Z4V2, is characterized by a higher content of phenolic compounds, flavonoids, proanthocyanidins, and anthraquinones. Finally, group 3, represented by samples Z2V2 and Z3V2, is very interesting because of their higher antiradical and reducing power. However, despite their good biological properties, the latter group is also characterized by a high content of biological compounds, but lower than that of group 2. This can be explained by the fact that the biological properties of a plant do not only depend on its bioactive compound content but are also closely related to the nature and the proportion of the bioactive compounds found in it. According to their higher antioxidant activities, samples Z2V2 and Z3V2 were used for the experimentation on rats to assess their effect on lipid profile management.

### 3.5. Effect of *Ipomea batatas* Leaf Powder on Body Weight Evolution

**Figure 2:**
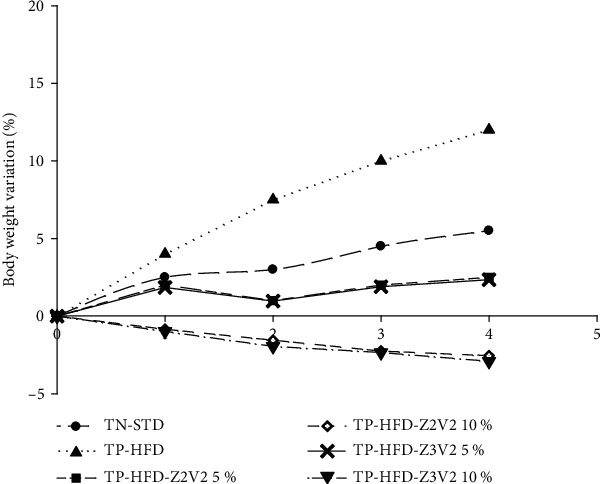
Changes in body weight of rats fed a high-fat diet supplemented or not with *Ipomea batatas* leaf powder. (Legend. TN-STD: the negative control standard died group receiving *ad libitum* a standard or normal diet; TP-HFD: the positive control high-fat diet group receiving *ad libitum* a high-fat diet; TP-HFD-Z2V2 5% and TP-HFD-Z2V2 10%: the positive control high-fat diet group receiving *ad libitum* a high-fat diet supplemented, respectively, with doses of 5 and 10% (*w*/*w*) of *I. batatas* leaf powder from Ngaoundere/white *I. batatas* IRAD-1112 Variety; TP-HFD-Z3V2 5% and TP-HFD-Z3V2 10%: the positive control high-fat diet group receiving *ad libitum* a high-fat diet supplemented, respectively, with doses of 5 and 10% (*w*/*w*) of *I. batatas* leaf powder from Foumban/white *I. batatas* IRAD-1112 Variety.

The ingestion of the high-fat diet resulted in a gradual increase in body weight with time, whereas weight gain was suppressed in a dose-dependent manner in all groups fed the *I. batatas* leaf powder supplemented HFD (Figure 2). A significant decrease in body weight was observed for the *I. batatas* leaf powder group (TP-HFD-Z2V2 5% and TP-HFD-Z3V2 5%) compared to the TP-HFD group feeding only with a high-fat diet (Figure 2). At the end of the experimentation, the weight gain of the *I. batatas* leaf powder group (TP-HFD-Z2V2 5% and TP-HFD-Z3V2 5%) was similar, and no significant difference was observed between both (2 and 1.9%, respectively). For the TP-HFD-10% group, a significant decrease in body weight (weight loss) was observed throughout the experimental period, and the final weight loss recorded was 2.9 and 3.2% of the initial weight, for TP-HFD-Z2V2 10% and TP-HFD-Z3V2 10%, respectively. It has been recommended that weight reduction programs should focus on achieving a modest weight loss of 7–10% of the initial weight. Therefore, *I. batatas* leaf powder could be used in weight reduction programs. The weight loss can be attributed to dietary fiber contains which were found in high amount in *I. batatas* leaf powder [33]. In previous studies [34, 35], evidence of a negative correlation between high fiber content and weight gain has been reported. Dietary fiber promotes the excretion of fat and alternatively as reported by Isken et al. [33], it is possible that lipid absorption in the small intestine was blocked by dietary fiber. These results are in agreement with those of Kurata et al. [20] who conducted a similar study by supplementing the high-fat diet of rats with 5% of *I. batatas* leaf powder collected in Japan. They found that 5% of the *I. batatas* leaf powder group showed significant suppression in final weight (390.6 ± 15.69 g) and adipose tissue weight (6.25 ± 0.95 g) compared to the positive control group feeding only a high-fat diet, and for which, the final weight and adipose tissue weight were 425.0 ± 24.97 g and 8.85 ± 1.32 g, respectively.

### 3.6. Effect of *Ipomea batatas* Leaf Powder on Serum Lipids Profile and Atherogenic Index

The total cholesterol, LDL-cholesterol, HDL-cholesterol, and total triglyceride concentrations (mg/dL) of the different groups of experimental rats are shown in Table 3. From this table, it can be observed that the final values of HDL-cholesterol concentration in rats fed with a hyperlipidemic diet TP-HFD (52.64 ± 0.24 mg/dL) are significantly (*p* < 0.05) lower than those of rats fed the normal diet TN-STD (64.40 ± 0.32 mg/dL), while the opposite is observed for the final values of serum total cholesterol, triglycerides, and LDL-cholesterol concentration between these two groups of rats. For rats in the TP-HFD group, the final values obtained are 190.22 ± 0.94 mg/dL, 154.56 ± 0.28 mg/dL, and 111.67 ± 0.86 mg/dL, for serum total cholesterol, triglycerides, and LDL cholesterol, respectively, while for rats in the TN-STD group, the final values obtained are 166.35 ± 1.00 mg/dL, 118.92 ± 0.28 mg/dL, and 97.33 ± 0.54 mg/dL, respectively. Numerous studies have shown that increased levels of serum total cholesterol, triglycerides, and LDL-cholesterol increase the risk of developing atherosclerosis and coronary heart disease [36, 37]. However, when *I. batatas* leaf powder was incorporated into the high-fat diet, significant reductions in serum total cholesterol, triglyceride, and LDL-cholesterol values were observed compared to the TP-HFD group. For rats in the TP-HFD-Z2V2-5% group, the final values obtained are 160.03 ± 0.36 mg/dL, 130.42 ± 0.33 mg/dL, and 90.17 ± 1.00 mg/dL, respectively. For rats in the HFD-Z2V2-10% group, the final values obtained are 153.15 ± 1.29 mg/dL, 122.98 ± 0.82 mg/dL, and 86.33 ± 1.15 mg/dL, respectively. These values were significantly lower than those of the TP-HFD-Z2V2 5% group. In addition, the supplementation with *I. batatas* leaf powder resulted in a significant increase in HDL-cholesterol in rats in the TP-HFD-Z2V2-5% and TP-HFD-Z2V2-10% groups (66.88 ± 2.46 mg/dL and 69.84 ± 0.13 mg/dL, respectively) compared to the TP-HFD group. The decrease in LDL-cholesterol and the increase in serum HDL cholesterol levels contribute to an antiatherogenic condition [37]. Similar results were observed in rats of the HFD-Z3V2-5% and HFD-Z3V2-10% groups, receiving a hyperlipidemic diet incorporated with 5 and 10% of the powder of the leaves of *I. batatas*, harvested in zone 3 and belonging to variety 2. The reduction in total serum cholesterol could be associated with the high dietary fiber content of *I. batatas* leaves. Several studies have shown that propionate generated from dietary fiber has hypocholesterolemic effects and compensates for the hypercholesterolemic effects of acetate production, which tends to increase total serum cholesterol via a mechanism probably involving hepatic lipogenesis [38, 39]. Several other mechanisms are involved in the reduction of total serum cholesterol; one of the best known is a decrease in the absorption of bile acids, which causes the elimination of steroids from the body through faecal excretion, an increase in cholesterol catabolism, an increase in the secretion of bile acids and a decrease in the secretion of cholesterol by lipoproteins [40]. In addition, several scientific studies have also shown that certain bioactive compounds present in plants can reduce serum cholesterol levels [41–46]. For example, saponins have cholesterol-lowering effects in animals and humans [47–50]. They have an amphiphilic structure and can bind to dietary cholesterol and/or bile acids. Saponins can prevent the absorption of cholesterol, interfere with its enterohepatic circulation, and increase its faecal excretion. The obvious presence of saponins in sweet potato leaves has been reported by Groppo et al. [6] and by Mbaeyi-Nwa and Emejulu [51]. In contrast to this study, Kurata et al. [20] found no significant difference in HDL-cholesterol and total cholesterol levels between the group of rats fed only a high-fat diet and the group of rats fed a high-fat diet supplemented with 5% *I. batatas* leaf powder. However, they found that, the plasma triglycerides level in the 5% *I. batatas* group was significantly lower (66.96 ± 35.29 mg/dL) and approximately 38% of that in the positive control group (178.63 ± 44.76 mg/dL). On the other hand, other research studies have reported similar results to the present study as they found that the aqueous extract of *I. batatas* leaves collected in Cameroon restored blood lipid levels to normal values in the group of rats fed a high-fat diet [52]. These authors also suggest that *I. batatas* leaves have hypolipidemic and antiatherosclerotic properties that justify their use in traditional medicine.

The TG/HDL-cholesterol ratio is linked to a risk factor for coronary heart disease, and the risk increases as this ratio also increase. This risk of cardiovascular disease is low when TG/HDL-cholesterol ratio is between −0.3 and 0.1, medium when TG/HDL-cholesterol ratio is between 0.1 and 0.24, and high when TG/HDL-cholesterol ratio is more than 0.24, as reported by Bo et al. [24]. From the observations made in Table 3, it is evident that the high-fat diet (0.47 ± 0.00) significantly increases the TG/HDL-cholesterol ratio compared to the normal diet (0.27 ± 0.00). For the different groups of rats fed a high-fat diet and supplemented with 10% of *I. batatas* leaf powder (TP-HFD-Z2V2-10% group and TP-HFD-Z3V2-10% group), no significant differences were found with an atherogenic index ranging from 0.25 ± 0.00 to 0.25 ± 0.01, respectively. However, these values were significantly low compared to the negative control groups and were also significantly low compared to rats fed a high-fat diet (TP-HFD) and to rats fed a high-fat diet and supplemented with 5% of *I. batatas* leaf powder (TP-HFD-Z2V2-5% group and TP-HFD-Z3V2-5% group, with an atherogenic index value of 0.29 ± 0.00 and 0.29 ± 0.00, respectively). These results indicate that *I. batatas* leaf powder contains certain biological compounds that prevent the deposition of cholesterol or lipids in certain organs (kidneys, heart, and liver) and arteries. However, in general, at the end of the experiment (4 weeks of feeding), all the different groups of rats can be classified according to the atherogenic index values obtained, as high-risk groups for cardiovascular disease with an atherogenic index greater than 0.24.

### 3.7. Effect of *Ipomea batatas* Leaf Powder on Serum aspartate aminotransferase (ASAT), Alanine Aminotransferase (ALAT), and Creatinine

The aspartate aminotransferase (ASAT) and alanine aminotransferase (ALAT) values in the liver were quantified to determine the degree of liver damage in experimental rats and the results are shown in Table 3. From this table, it can be observed that a high-fat diet leads to a significant increase in the values of the liver parameters analyzed, except for creatinine level. Thus, the TP-HFD group has an ALAT, ASAT values, and creatinine concentration of 29.86 ± 0.83 UI/L, 37.83 ± 0.43 UI/L, and 12.70 ± 0.47 *μ*mol/L, respectively. While the TN-STD group has an ALAT, ASAT values, and creatinine concentration of 16.06 ± 0.83 UI/L, 33.20 ± 0.59 UI/L, and 12.06 ± 0.78 *μ*mol/L, respectively. Compared to the TP-HFD group, supplementation with *I. batatas* leaf powder significantly reduced the ALAT value in rats in the TP-HFD-Z2V2 and TP-HFD-Z3V2 groups. ALAT values ranged from 15.44 ± 0.34 UI/L to 26.28 ± 0.87 UI/L. Groups TP-HFD-Z2V2-10% and TP-HFD-Z3V2-10% had the lowest values (15.44 ± 0.34 UI/L and 15.68 ± 0.29 UI/L, respectively), which were not significantly different from the TN-STD group (16.06 ± 0.83 UI/L). However, compared to the TN-STD group (33.20 ± 0.59 UI/L), the ASAT values (38.25 ± 0.73 UI/L to 41.37 ± 0.55 UI/L) remained significantly higher in all groups receiving a hyperlipidemic diet supplemented with *I. batatas* leaf powder. No significant difference was observed for TP-HFD-Z3V2-10% (38.27 ± 0.93 UI/L) and TP-HFD-Z2V2-10% (38.25 ± 0.73 UI/L) groups compared to the TP-HFD group (37.83 ± 0.43 UI/L). Regarding creatinine levels, the groups of rats fed a high-fat diet and supplemented with 5 and 10% of *I. batatas* leaf powder had a significantly lower creatinine level (9.73 ± 0.30 *μ*mol/L to 11.19 ± 0.34 *μ*mol/L) than the TN-STD (12.06 ± 0.78 *μ*mol/L) and TP-HFD (12.70 ± 0.47 *μ*mol/L) groups. These results show that supplementation of a diet with 10% of *I. batatas* leaf powder reduces the degree of liver damage and normalizes serum creatinine levels in rats fed a high-fat diet, indicating its protective effect on renal glomerular filtration capacity, as reported by Suanarunsawat and Songsak [53].

## 4. Conclusion

This study reveals the high fiber content in *Ipomea batatas* leaf powders, which also contain phytochemical compounds such as flavonoids, proanthocyanidins, and anthraquinones. *I. batatas* leaf powders also showed good antiradical and reducing activity, particularly samples Z3V2 and Z2V2 of variety 2 collected, respectively, in West and Adamaoua. In rats fed with a high-fat diet, supplementation with 10% *I. batatas* leaf powder was effective in reducing the levels of triglycerides, total cholesterol, and LDL-cholesterol as well as ALAT activity and creatinine concentration. The results of this study suggest the use of *I. batatas* leaves as a phytomedicine in the treatment of cardiovascular diseases. In the further development of this study, it would be interesting to identify, using analytical techniques, the phytochemical compounds endowed with these properties.

## Figures and Tables

**Table 1:** Composition of standard and high-fat diets as proposed by Murase et al. (2002).

Ingredients	Standard diet (g/100 g)	High-fat diet (g/100 g)
Corn starch	70	40
Casein	21	21
Soybean oil	5	5
Palm oil	00	30
Mineral mix	3	3
Vitamin mix	1	1

**Table 2:** Total fiber content, phytochemical compound, and antioxidant activity of *Ipomea batatas* leaf powder.

Samples	Bioactive compounds					Antioxidant activity		
	Total fiber (mg/100gDW)	Total phenolics (mgGAE/100gDW)	Flavonoids (mgCE/100gDW)	Proanthocyanidins (mgCE/100gDW)	Anthraquinones (mgEC/100gDW)	FRAP (mg AAE/gDW)	TAC (mg AAE/gDW)	DPPH/IC_50_ (mg/mL)
Z1V1	9.19 ± 0.06^d^	659.45 ± 0.82^b,c^	288.14 ± 0.06^b^	175.57 ± 0.35^b,c^	325.72 ± 3.06^a^	3.29 ± 0.27^a,b^	19.00 ± 0.88^a^	2.90 ± 0.04^bc^
Z2V1	9.51 ± 0.06^e^	659.02 ± 1.16^a,b,c^	289.06 ± 1.01^c^	175.27 ± 0.05^a^	325.41 ± 1.53^a^	3.02 ± 0.13^a^	19.26 ± 0.51^a^	2.94 ± 0.07^bc^
Z3V1	8.86 ± 0.05^c^	657.76 ± 0.31^a^	282.25 ± 0.12^a^	175.40 ± 0.06^a,b^	324.39 ± 1.53^a^	3.11 ± 0.08^a^	20.08 ± 3.77^a,b^	3.06 ± 0.06^c^
Z4V1	8.15 ± 0.02^a^	659.38 ± 0.54^b,c^	289.13 ± 0.96^c^	175.83 ± 0.09^d,e^	325.89 ± 2.25^a^	3.11 ± 0.39^a^	20.20 ± 1.82^a,b^	3.08 ± 0.08^c^
Z1V2	12.42 ± 0.09^h^	660.02 ± 0.82^b,c^	325.05 ± 0.00^e^	176.04 ± 0.06^e^	326.38 ± 3.05^a^	3.70 ± 0.21^c^	20.13 ± 1.92^a,b^	2.31 ± 0.03^b^
Z2V2	9.87 ± 0.08^f^	659.06 ± 0.15^a,b,c^	324.03 ± 0.01^d^	175.75 ± 0.18^c,d^	325.40 ± 1.53^a^	3.83 ± 0.09^c,d^	23.02 ± 1.17^b^	1.66 ± 0.04^a^
Z3V2	10.01 ± 0.05^g^	658.68 ± 0.94^a,b^	323.22 ± 0.20^d^	175.65 ± 0.08^b,c,d^	324.05 ± 2.00^a^	4.10 ± 0.09^d^	23.48 ± 2.75^b^	1.58 ± 0.02^a^
Z4V2	8.62 ± 0.07^b^	660.10 ± 1.00^c^	325.03 ± 0.02^e^	176.03 ± 0.06^e^	326.72 ± 2.52^a^	3.67 ± 0.29^b,c^	19.11 ± 1.24^a^	1.90 ± 0.05^ab^

Values with different superscripts in the same column are statistically different (*p* < 0.05); mean values ± standard deviation values; *n* = 3. (Legend. Z1V1: yellow *I. batatas* IRAD-Tib1 collected from Garoua; Z2V1: yellow *I. batatas* IRAD-Tib1 collected from Ngaoundere; Z3V1: yellow *I. batatas* IRAD-Tib1 collected from Foumban; Z4V1: yellow *I. batatas* IRAD-Tib1 collected from Bafia; Z1V2: white *I. batatas* IRAD-1112 collected from Garoua; Z2V2: white *I. batatas* IRAD-1112 collected from Ngaoundere; Z3V2: white *I. batatas* IRAD-1112 collected from Foumban; Z4V2: white *I. batatas* IRAD-1112 collected from Bafia; FRAP: ferric reducing antioxidant power; TAC: total antioxidant capacity; DPPH/IC_50_: concentration of the extract that allows 50% of the DPPH free radical to be trapped).

**Table 3:** Changes in serum lipid profile, aspartate aminotransferase (ASAT), alanine aminotransferase (ALAT), and serum creatinine in rats fed a high-fat diet supplemented or not with *I. batatas* leaf powder.

Samples	Lipid profile and atherogenic index (AI)					Hepatic and cardiac parameters		
	TG (mg/dL)	T Chl (mg/dL)	HDL-cholesterol (mg/dL)	LDL-cholesterol (mg/dL)	AI	ASAT (UI/L)	ALAT (UI/L)	Creatinine (*μ*mol/L)
TN-STD	118.92 ± 0.28^a^	166.35 ± 1.00^c^	64.40 ± 0.32^b^	97.33 ± 0.54^c^	0.27 ± 0.00^b^	33.20 ± 0.59^a^	16.06 ± 0.83^a^	12.06 ± 0.78^c^
TP-HFD	154.56 ± 0.28^e^	190.22 ± 0.94^d^	52.64 ± 0.24^a^	111.67 ± 0.86^d^	0.47 ± 0.00^d^	37.83 ± 0.43^b^	29.86 ± 0.83^c^	12.70 ± 0.47^c^
TP-HFD-Z2V2-5%	130.42 ± 0.33^c^	160.03 ± 0.36^b^	66.88 ± 2.46^c^	90.17 ± 1.00^b^	0.29 ± 0.02^c^	41.19 ± 0.34^c^	25.91 ± 0.46^b^	11.08 ± 0.48^b^
TP-HFD-Z2V2-10%	122.98 ± 0.82^b^	153.15 ± 1.29^a^	69.84 ± 0.13^d^	86.33 ± 1.15^a^	0.25 ± 0.00^a^	38.25 ± 0.73^b^	15.44 ± 0.34^a^	9.73 ± 0.30^a^
TP-HFD-Z3V2-5%	132.67 ± 1.70^d^	161.26 ± 0.62^b^	67.55 ± 1.27^c^	91.33 ± 0.54^b^	0.29 ± 0.01^c^	41.37 ± 0.55^c^	26.28 ± 0.87^b^	11.19 ± 0.34^b^
TP-HFD-Z3V2-10%	124.19 ± 0.77^b^	154.56 ± 1.25^a^	70.28 ± 1.87^d^	87.00 ± 1.28^a^	0.25 ± 0.01^a^	38.27 ± 0.93^b^	15.68 ± 0.29^a^	9.75 ± 0.59^a^

Values with different superscripts in the same column are statistically different (*p* < 0.05); mean values ± standard deviation values; *n* = 3. (Legend. TN-STD: the negative control standard died group receiving *ad libitum* a standard or normal diet; TP-HFD: the positive control high-fat diet group receiving *ad libitum* a high-fat diet; TP-HFD-Z2V2 5% and TP-HFD-Z2V2 10%: the positive control high-fat diet group receiving *ad libitum* a high-fat diet supplemented, respectively, with doses of 5 and 10% (*w*/*w*) of *I. batatas* leaf powder from Ngaoundere/white *I. batatas* IRAD-1112 Variety; TP-HFD-Z3V2 5% and TP-HFD-Z3V2 10%: the positive control high-fat diet group receiving *ad libitum* a high-fat diet supplemented, respectively, with doses of 5 and 10% (*w*/*w*) of *I. batatas* leaf powder from Foumban/white *I. batatas* IRAD-1112 Variety; TG: triglycerides; T Chl: total cholesterol).

## Data Availability

The data used to support the findings of this study are available from the corresponding author upon request.

## Abbreviation

Z1: North (Garoua)
Z2: Adamawa (Ngaoundere)
Z3: West (Foumban)
Z4: Center (Bafia)
V1: Yellow (IRAD-Tib1)
V2: White (IRAD-1112).
